# Professional Quality of Life and Perceived Stress in Health Professionals before COVID-19 in Spain: Primary and Hospital Care

**DOI:** 10.3390/healthcare8040484

**Published:** 2020-11-13

**Authors:** Ángela María Ortega-Galán, María Dolores Ruiz-Fernández, María-Jesús Lirola, Juan Diego Ramos-Pichardo, Olivia Ibáñez-Masero, José Cabrera-Troya, Virginia Salinas-Pérez, Piedras Alba Gómez-Beltrán, Elia Fernández-Martínez

**Affiliations:** 1 Nursing Department, University of Huelva, 21071 Huelva, Spain; angela.ortega@denf.uhu.es (Á.M.O.-G.); juan.ramos@denf.uhu.es (J.D.R.-P.); olivia.ibanez@denf.uhu.es (O.I.-M.); albi71@hotmail.com (P.A.G.-B.); elia.fernandez@denf.uhu.es (E.F.-M.); 2 Department of Nursing, Medicine and Physiotherapy, Faculty of Health Sciences, University of Almeria, 04120 Almeria, Spain; 3 Faculty of Education Sciences, University of Almeria, 04120 Almeria, Spain; 4 Health Management Area Seville South, 41014 Seville, Spain; deliatadeo@gmail.com; 5 Málaga-Guadalhorce Sanitary District, 29009 Malaga, Spain; salinasperezforero@gmail.com

**Keywords:** compassion fatigue, compassion satisfaction, burnout, empathy, post-traumatic stress, health professional

## Abstract

This study aimed to analyze the professional quality of life and the perceived stress of health professionals before COVID-19 in Spain, in primary and hospital care professionals. A cross-sectional observational study on health professionals working in health centers during the health crisis caused by COVID-19 was conducted. Professional Quality of Life (ProQoL) and Perceived Stress (PSS-14) were measured, along with socio-demographic and labor variables through an online questionnaire. A descriptive and correlation analysis was performed. A total of 537 professionals participated, both in hospital care (54.7%) and in primary care (45.3%). There was a predominance of medium Compassion Satisfaction, high Compassion Fatigue and medium Burnout. Mean scores for compassion fatigue and compassion satisfaction were slightly higher in primary care, while burnout was higher in hospital care. When primary care participants were grouped by profession, significant differences were found in relation to perceived stress and to the three subscales of professional quality of life. In hospital care, the differences were observed when comparing compassion fatigue and perceived stress by gender. In addition, with respect to Burnout it was carried out by type of contract and shift and in relation to perceived stress grouped by sex, contract and profession. The COVID-19 health crisis has had an impact on mental health and the quality of professional life of health professionals. There is a need to implement long-term contingency programs aimed at improving the emotional well-being of health service professionals.

## 1. Introduction

In recent years, the pandemic as a result of COVID-19 has been one of the most serious situations for humanity [1] In Spain, the incidence of COVID-19 has been particularly complex with a high number of people affected and a high mortality rate [2]. The serious consequences produced by the disease imply people requiring hospitalization and special care [3].

One of the groups in which this health crisis has had the greatest impact has been health professionals [4]. They have been involved in an overwhelming situation at the professional level with the increase in demand, the overload of work, the risk of infection, the possibility of transmission to their families, confinement and in many cases voluntary isolation, among other circumstances [4,5]. The effect has been on both primary and specialized care professionals, although with slightly different peculiarities. Thus, new and important ethical challenges have arisen regarding the prioritization of treatment, protective equipment and tests; the impact of COVID-19 strategies on patients with other processes, resuscitation decisions, the option for telemedicine in the primary health care setting and the crisis in nursing homes [6,7].

This scenario can generate in health professionals a series of psychological symptoms such as fear, insecurity and anxiety [4,5,8,9]. A syndrome closely related to traumatic and complex situations in the work context of health professionals is Compassion Fatigue (CF) [10]. Stamm [11] included it within a wider concept, the Professional Quality of Life, in conjunction with Burnout (BO) and Compassion Satisfaction (CS). CF or secondary traumatic stress is the cost of being concerned about others or about their emotional pain, which results from the desire to relieve the suffering of others [10]. Closely related to this is BO, which involves emotional exhaustion, depersonalization and lack of personal fulfillment at work as a result of continued exposure to job stressors [12,13]. On the contrary, CS acts as a protective factor, meaning that health professionals can experience this feeling when doing their job well, furthermore including satisfaction in their relationship with their colleagues and the feeling the social value of the work done [10].

CF has been studied in certain healthcare settings such as emergency services, critical care and primary care [12,14,15]. In these settings, it has been shown that the quality of professional life is altered and very high levels of CF occur among healthcare professionals. Recently these variables (CS, CF, and BO) have been studied to understand how doctors and nurses have been affected by the pandemic [16]. Notwithstanding, in this scenario of health crisis in which health professionals have been exposed to high levels of emotional tension, stress and suffering [17], it is not known how this situation has affected the quality of professional life and the perceived stress in different healthcare environments in our country (Hospital and primary care) in addition to taking into account a broader spectrum of health professionals, including doctors, nurses and technicians. It is necessary to know this situation in order to implement contingency programs that improve or maintain the quality of professional life and respond to emotional distress [12,18]. Therefore, the objective of this research was to analyze the professional quality of life and the perceived stress of health professionals in light of COVID-19 in Spain, according to the level of care (hospital and primary care).

## 2. Materials and Methods

### 2.1. Design

**Figure 1:**
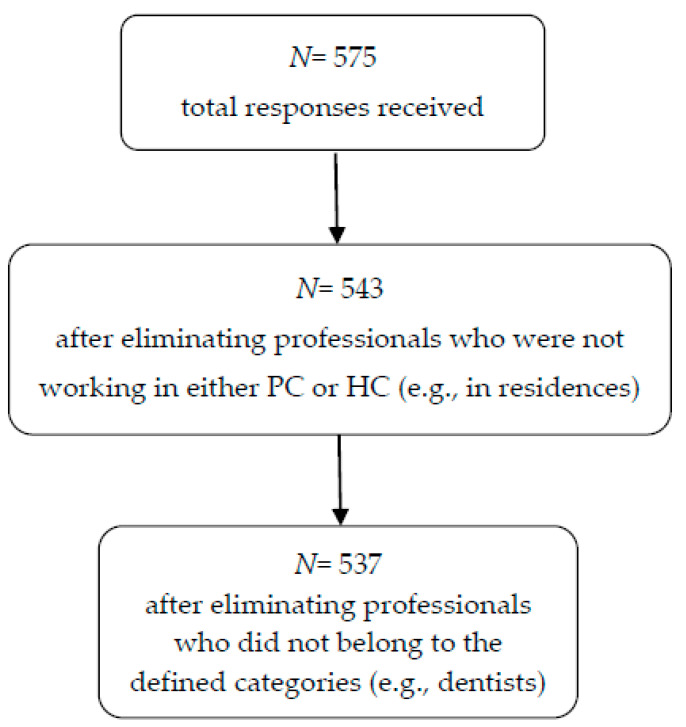
Flowchart of professionals included in the study.

A transversal observational study was carried out with a sample of 537 health professionals (doctors, nurses and health technicians) who met the following inclusion criteria: professionals who were in an active situation, in care services of the Spanish National Health System and who worked in direct contact with patients. Excluded were professionals who carried out exclusively teaching or management functions or worked in services where there was no direct care with patients (i.e., laboratory services, sterilization, etc.). Figure 1 shows the sample selection flow and the process used to reach the final sample.

### 2.2. Instruments

The professional quality of life was evaluated with the Professional Quality of Life Scale (ProQoL) [11], translated and used in professionals with the Spanish health context exposed to situations of stress and suffering [12,19]. It consists of 30 items with a Likert type score of 6 points (from 0 = never to 5 = always). The questionnaire is composed of three subscales: Compassion Fatigue (10 items), Compassion Satisfaction (10 items) and Burnout (10 items). The higher the score in each of the dimensions, the higher the level of CF, CS and BO, respectively. The scores can be categorized in each of the subscales into CF (<8 low; 9–17 medium; >17 high); CS (<33 low; 34–41 medium; >42 high); and BO (<18 low; 19–26 medium; >27 high). The Spanish version of ProQoL has shown a Cronbach alpha of 0.782 in CF, of 0.774 in CS and 0.537 for BO, respectively [19].

Perceived stress was evaluated with the Perceived Stress Scale (PSS-14) [20] which measures the level of perceived stress in the last month, under certain life circumstances. It consists of 14 items with a Likert type score that varies from 0 (never) to 4 (very often). The higher the score, the greater the perceived stress. The reliability in the Spanish population was a Cronbach alpha of 0.81 [21].

In addition, sociodemographic and labor context data were collected through a questionnaire designed ad hoc: age, sex, marital status, level of care, type of labor contract, work shift.

### 2.3. Procedure

The data collection started on 30 March until 16 April 2020. This period coincided with the period of maximum incidence of cases and mortality by COVID-19, in Spain. Due to the conditions of social distancing caused by confinement on those dates, an online questionnaire was designed for the collection of data with the instruments described above. The estimated time of completion of the questionnaire was 15 min. The link to respond was disseminated to health professionals through the web page and social networks of the research group [http://cuidadoscompasion.es/]. Participation was totally voluntary and anonymous.

### 2.4. Ethical Considerations

The study obtained permission from the research ethics committee of the Almería Center (CEI-27/9/2017). The participants were informed about the aim of the study. They were asked for informed consent for their participation. The ethical principles of the Declaration of Helsinki were respected. The confidentiality and anonymity of the participants complied with the national regulations regarding the protection of personal data (Organic Law 3/2018 on the protection of personal data and the guarantee of digital rights).

### 2.5. Data Analysis

The data were imported into an Excel worksheet from the Microsoft Office package and then analyzed using the SPSS v23 statistical package for social sciences. First, descriptive statistics of the measured variables were calculated. Subsequently, different analyses were carried out to compare the scores obtained in the different scales (CS, CF, BO and PSS-14) according to sex and other dichotomous variables, using the Student t-test. On the other hand, a one-factor ANOVA was carried out for the analysis of the differences found in the politomic socio-demographic and labor variables. For the comparison of groups according to ordinal variables, the Mann–Withney U test of linear trend was used. Finally, Pearson’s correlation test was applied for the relations between the different measured constructs. The suitability of the statistical tests used was verified in all cases. The significance level was set at *p* < 0.05.

## 3. Results

### 3.1. Socio-Demographic Characteristics of the Participants

A total of 537 health professionals participated, with an average age of 46.74 ± 10.08 years. The sample size was calculated based on the number of health professional who worked at the Andalusian Public Health System in 2019 [22] (*n* = 71,827), obtaining a 97% confidence level and 4% margin of error. At the time of the study, 45.3% (*n* = 243) worked in primary care (PC) and 54.7% (*n* = 294) in hospital care (HC). Table 1 shows the socio-demographic and occupational characteristics of the participants, according to their level of care. In both PC and HC, the majority of the sample were women, married or in a domestic partnership, and professional nurses. In HC, most of the sample had a non-permanent contract and a day shift with nights or guards. In PC most were professionals with a permanent contract and with a regular day shift. When comparing socio-demographic and labor characteristics according to the level of care, statistically significant differences were found with respect to marital status, type of contract and work shift.

### 3.2. Professional Quality of Life and Perceived Stress in PC and HC Professionals

The mean score in CS and BO was higher in HC than PC, while the mean score in CF and perceived stress was higher in PC than in HC. When interpreting the score of each one of the subscales of the Professional Quality of Life questionnaire, in the three categories advised by the literature (low/medium/high), it was identified that in both HC and PC there was a higher percentage in medium CS, high CF and medium BO. If we compare both levels of attention, there was a higher CS and medium CF in PC than in HC, HC being where a high level of BO was observed. Nevertheless, no statistically significant differences were found when comparing the prevalence of BO categories between PC and HC professionals (Table 2).

Table 3 shows the professional quality of life and perceived stress, according to socio-demographic and occupational characteristics, both in primary care and in hospital. When comparing the average CS score in HC professionals, no differences were found by comparing them by the categories of sociodemographic and labor variables. Nonetheless, in the group practicing PC, a significant higher mean score was identified in non-permanent professionals compared to permanent professionals (*p* = 043), and significant differences were also found in the scores of nurses and doctors (*p* < 0.01), and health technicians and doctors (*p* < 0.01). From these, the highest scores were those of PC health technicians and the lowest were those of PC doctors. Regarding the mean scores of the CF subscale, the mean was significantly higher in women than in men, although statistically significant differences were only identified in this respect in HC professionals (*p* = 0.028). However, significant differences were identified in relation to this scale in the PC group, depending on the profession, with doctors scoring higher than nurses (*p* < 0.01) and health technicians (*p* = 0.031). In the BO subscale, significant differences were found in HC professionals when comparing professionals according to the shift they had. A higher average significant score was identified in professionals with a regular night shift, compared to those with a day shift without nights (*p* = 0.015), between regular morning shifts and rotating shifts without nights (*p* = 0.038), and between rotating shifts with and without nights (*p* = 0.012). The highest average score corresponded to the regular night shift and the lowest to the day shift without nights and the regular morning shift. On the other hand, for the same scale in PC professionals, the following were found in all comparisons between pairs of professions: medicine with nursing (*p* < 0.01), nursing with health technicians (*p* = 0.032), and medicine with health technicians (*p* < 0.01). In this sense, the highest average figure corresponded to the medical profession and the lowest average to health technicians.

In relation to perceived stress, a significant higher average score was identified in women compared to men in HC professionals (*p* < 0.01). Regarding the profession, in HC the highest average significant figure was for nurses, which showed statistically significant differences when compared to the average for technicians (*p* = 0.014). In the case of PC, the technicians obtained statistically lower figures than both doctors (*p* < 0.01) and nurses (*p* = 0.018), and in contrast to HC, in PC the highest average of perceived stress corresponded to doctors.

### 3.3. Correlations between the Professional Quality of Life Subscales and the Perceived Stress Scale

The results of the analysis of correlation between CS, CF, BO and perceived stress are shown in Table 4. In the total of participants and in the subsamples of PC and HC, CS was significantly and inversely related to BO and perceived stress. A direct and significant correlation was obtained between CF and BO, CF and perceived stress, as well as for BO and perceived stress, both in the total sample and in the professionals of PC and HC.

## 4. Discussion

The results of this research report that CF and BO levels are medium to high in PC and HC professionals. The point in time when the information was collected in Spain was in the weeks of greatest severity, corresponding to the first wave of the pandemic. Despite the significance and concern of this finding, it is necessary to know that the pre-pandemic situation around professionals was already alarming. We can find numerous studies that evidence high rates of BO, CF and perceived stress among health professionals around the world [23,24].

Secondly, in this study no significant differences were found between the levels of care (PC and HC) in the dimensions of ProQoL (CS, CF and BO), or in terms of perceived stress. Nevertheless, in previous studies it was found that the average score in CF is significantly higher in primary care than in hospital care [12]. The authors of this study explain this by the lack of resources at the primary health care level. During this time, and taking into account that the data were taken during the worst period in Spain of the COVID-19 pandemic, it is reasonable to expect that they have increased in HC and have equalized to the levels of PC. The deficit of resources was exponential at both levels of care, increasing the risk of suffering from compassion fatigue syndrome among the different profiles of health professionals [8].

In relation to gender, the results indicate that women in specialized care have greater compassion fatigue than men and more perceived stress, which is consistent with previously published studies [12]. This may be due to the high level of feminization of health professionals, which means that frontline care is mainly provided by women [25]. In addition, it may be influenced by the social role that women play with respect to caregiving [26]. Notwithstanding, the influence of gender on the phenomenon of compassion fatigue in the health care professions is not sufficiently explored and should be one of the areas for further study in the future.

The fact that primary care doctors are the professional profile most affected by the pandemic is one of the relevant findings. They can be seen to have the highest levels of CF, the highest perceived stress and the lowest CS. The lack of planning for potential health emergencies has led to a substantial change in healthcare at all levels [27] and especially in PC. As a result, professionals in this first line of action have become more vulnerable to moral damage and compassion fatigue [28].

On the other hand, the presence of moderate and high levels of CF, moderate and high levels of BO, and moderate and high levels of CS may seem a paradox. According to these data, high levels of CF and CS can be reconciled at certain times, a question that on the other hand, contradicts the structure of the questionnaire used. This could be explained by the difficulties found in the ProQoL questionnaire expressed in some works that have questioned whether its dimensions effectively measure the concepts from which they were originated [29,30]. Furthermore, it could be explained by the exceptional moment that professionals live in which it is possible to feel tired, worn out and at the same time, feel a great satisfaction for what their work means, which is born from the genuine desire to alleviate suffering. In this time of pandemic, professionals have been able to recover their values and motivation for their work, although at the same time, they have been able to feel tremendously impacted by the scenario of enormous suffering that they have to face on a daily basis.

### Limitations and Strengths

The main limitation of this study is that the data have been obtained from a cross-sectional design study, which does not allow evaluation of the evolution over time of the study variables. The online collection of data can also be considered a weakness and implies a selection bias, although due to health circumstances it could not be done in a physical presence. In addition, the social desirability bias in the completion of questionnaires is not excluded in this research. Thus, given the limitations of the cross-sectional design, selection bias, and social desirability bias, it should be noted that the generalizability of the results is low due to the aforementioned limitations. As a strength, it should be noted that it is a first approximation to the professional quality of life and perceived stress from a joint perspective, which includes primary care and specialized care professionals.

## 5. Conclusions

Nevertheless, it is necessary to emphasize that it is very important to pay attention to the mental health situation of health professionals, especially in this time of pandemic [31,32]. Therefore, urgent and appropriate support is needed for all health workers worldwide who are working hard to control the outbreak of COVID-19 [33] and to establish early strategies that aim to prevent and treat indirect trauma [26]. This preventive approach is particularly important to avoid an increase in mental health problems among professionals at all levels [34]. In addition, programs for stress reduction, emotional regulation, and cultivation of compassion based on full attention will need to be established [35] for professionals affected by this compassion fatigue syndrome [36,37].

## Figures and Tables

**Table 1:** Sociodemographic and labor characteristics: primary and hospital care.

Characteristics	Variables	HC	PC	Mann–Whitney U
Age (years)		44.29 ± 9.71	49.70 ± 9.74	26,999.50
Gender	Female	226 (76.9%)	178 (73.3%)	
	Male	68 (23.1%)	65 (26.7%)	
Marital status	Married/Domestic partner	193 (65.6%)	199 (8.6%)	23,400.50 ***
	Single	35 (11.9%)	21 (8.6%)	
	Widowed/Divorced	66 (22.4%)	23 (9.5%)	
Profession	Nurse	215 (73.1%)	160 (65.8%)	25,772.50
	Doctor	43 (14.6%)	65 (26.7%)	
	Technicians	36 (12.2%)	18 (7.4%)	
Type of employment contract	Permanent	137 (46.6%)	153 (63%)	22,179.00 ***
	Temporary	157 (53.4%)	90 (37%)	
Work shift	Rotating without nights	47 (16%)	3 (1.2%)	15,159.50 ***
	Daytime with nights/guards	162 (55.1%)	93 (38.3%)	
	Constant day shift	79 (26.9%)	147 (60.5%)	
	Constant night shift	6 (2%)	0%	

HC = Hospital Care; PC = Primary Care; *** *p* < 0.001.

**Table 2:** Professional quality of life and perceived stress: hospital care and primary care.

Variables	Variables	HC		PC		Mann–Whitney U
		Mean ± SD	N (%)	Mean ± SD	N (%)	
CS		39.58 (6.25)		38.98 (6.64)		25,450.50
	Low		42 (14.3%)		42 (17.2%)	
	Medium		127 (43.2%)		106 (43.6%)	
	High		125 (42.5%)		95 (39.1%)	
CF		19.64 (7.59)		19.87 (7.75)		27,698.00
	Low		21 (7.1%)		14 (5.8%)	
	Medium		84 (28.6%)		14 (30.5%)	
	High		189 (64.3%)		155 (63.8%)	
BO		24.70 (5.96)		24.43 (6.05)		27,094.00
	Low		45 (15.3%)		45 (18.5%)	
	Medium		141 (48%)		116 (47.7%)	
	High		108 (36.7%)		82 (33.7%)	
PSS-14		25.98 (9.16)		26.44 (9.21)		

CS = Compassion Satisfaction; CF = Compassion Fatigue; BO = Burnout; PSS-14 = Perceived Stress; HC = Hospital Care; PC = Primary Care.

**Table 3:** Professional quality of life and perceived stress, according to socio-demographic and occupational characteristics: primary and hospital care.

Characteristcs	Variables	CS				CF				BO				PSS-14			
Variables	Variables	HC		PC		HC		PC		HC		PC		HC		PC	
		Mean ± SD	*p*-Value	Mean ± SD	*p*-Value	Mean ± SD	*p*-Value	Mean ± SD	*p*-Value	Mean ± SD	*p*-Value	Mean ± SD	*p*-Value	Mean ± SD	*p*-value	Mean ± SD	*p*-Value
Gender	Female	39.52 ± 6.52	0.767 ^a^	39.19 ± 6.03	0.475 ^a^	20.18 ± 7.51	0.028 ^a,^*	20.21 ± 7.81	0.252 ^a^	24.95 ± 5.80	0.191 ^a^	24.77 ± 6.04	0.151 ^a^	27.02 ± 9.17	0.000 ^a,^**	27.06 ± 6.40	0.130 ^a^
	Male	39.78 ± 5.34		38.40 ± 8.12		17.87 ± 7.67		18.92 ± 7.59		23.87 ± 6.46		23.51 ± 6.04		22.54 ± 8.33		24.75 ± 11.03	
Marital Status	Married	39.25 ± 5.56	0.777 ^b^	39.01 ± 6.59	0.960 ^b^	20.13 ± 7.55	0.218 ^b^	19.76 ± 7.67	0.651 ^b^	24.83 ± 5.85	0.603 ^b^	24.25 ± 5.84	0.168 ^b^	26.32 ± 8.66	0.689 ^b^	26.07 ± 9.36	0.414 ^b^
	Single	38.97 ± 7.55		39.10 ± 6.73		19.57 ± 7.33		19.38 ± 8.27		23.74 ± 6.24		23.71 ± 6.91		25.46 ± 10.00		28.14 ± 9.32	
	Widowed/Divorced	39.58 ± 7.42		38.61 ± 7.23		18.24 ± 7.82		21.26 ± 8.24		24.82 ± 6.19		26.65 ± 6.83		25.29 ± 10.18		28.09 ± 7.74	
Contract	Permanent	39.45 ± 6.57	0.742 ^a^	38.32 ± 6.91	0.043 ^a,^*	19.01 ± 7.46	0.186 ^a^	20.01 ± 7.74	0.718 ^a^	23.96 ± 5.97	0.049 ^a,^*	24.41 ± 6.04	0.928 ^a^	24.34 ± 9.01	0.004 ^a,^**	26.18 ± 9.34	0.571 ^a^
	Temporary	39.69 ± 6.00		40.10 ± 6.03		20.19 ± 7.70		19.63 ± 7.80		25.34 ± 5.91		24.48.611		27.42 ± 9.08		26.88 ± 9.04	
Profession	Nurse	39.77 ± 6.32	0.261 ^b^	39.82 ± 5.61	0.001 ^b,^**	19.82 ± 7.62	0.359 ^b^	18.99 ± 6.80	0.005 ^b,^**	24.64 ± 5.84	0.324 ^b^	23.99 ± 5.63	0.001 ^b,^**	26.51 ± 9.01	0.048 ^b,^*	26.19 ± 8.54	0.005 ^b,^**
	Doctor	38.16 ± 6.54		36.32 ± 8.12		20.16 ± 7.31		22.52 ± 9.31		25.77 ± 6.47		26.51 ± 6.63		26.28 ± 9.47		28.62 ± 10.66	
	Health technician	40.14 ± 5.45		41.11 ± 6.84		17.97 ± 7.83		18.11 ± 7.70		23.78 ± 6.06		20.83 ± 5.22		22.47 ± 9.19		20.83 ± 6.82	
Work shift	Dayshift rotating	40.34 ± 5.47	0.661 ^b^	39.00 ± 1.00	0.999 ^b^	17.23 ± 7.28	0.113 ^b^	16.33 ± 4.04	0.775 ^b^	22.60 ± 4.75	0.023 ^b,^*	24.33 ± 1.16	0.470 ^b^	23.57 ± 9.06	0.170 ^b^	24.00 ± 3.00	0.620 ^b^
	Nights rotating	39.67 ± 6.46		39.00 ± 6.69		20.16 ± 7.64		20.51 ± 7.19		25.07 ± 6.11		24.78 ± 6.42		25.99 ± 8.88		27.12 ± 8.85	
	Constant day shift	39.05 ± 6.26		38.97 ± 6.64		19.86 ± 7.57		19.54 ± 8.14		24.86 ± 5.97		24.21 ± 5.89		27.32 ± 9.80		26.06 ± 9.52	
	Constant night shift	39.17 ± 7.39				21.67 ± 7.37				28.83 ± 7.20				27.17 ± 6.80			

CS = Compassion Satisfaction; CF = Compassion Fatigue; BO = Burnout; PSS-14 = Perceived Stress; HC = Hospital Care; PC = Primary Care; ^a^ Student *t*-Test for independent samples; ^b^ one-factor Banova; ** *p* < 0.01; * *p* < 0.05.

**Table 4:** Correlations between Quality of Life and Perceived Stress.

Variables	All professionals				PC Professionals				HC Professionals			
	1	2	3	4	1	2	3	4	1	2	3	4
1. CS		−0.403 **	−0.563 **	−0.454 **		-434 **	−0.568 **	−0.499 **		−0.375 **	−0.563	−0.413
2. CF			0.720 **	0.712 **			0.714 **	0.687 **			0.726 **	0.733 **
3. BO				0.620 **				0.633 **				0.611 **
4. PSS-14				-								

CS = Compassion Satisfaction; CF = Compassion Fatigue; BO = Burnout; PSS-14 = Perceived Stress; HC = Hospital Care; PC = Primary Care; ** *p* < 0.01.

## References

[1] Rothan H.A., Byrareddy S.N. (2020). The epidemiology and pathogenesis of coronavirus disease (COVID-19) outbreak. J. Autoimmun.

[2] Tejedor S., Cervi L., Tusa F., Portales M., Zabotina M. (2020). Information on the COVID-19 Pandemic in Daily Newspapers’ Front Pages: Case Study of Spain and Italy. Int. J. Env. Res. Public Health.

[3] Lai C.C., Wang C.Y., Wang Y.H., Hsueh S.C., Ko W.C., Hsueh P.R. (2020). Global epidemiology of coronavirus disease 2019 (COVID-19): Disease incidence, daily cumulative index, mortality, and their association with country healthcare resources and economic status. Int. J. Antimicrob. Agents.

[4] Xiang Y.T., Yang Y., Li W., Zhang L., Zhang Q., Cheung T., Ng C.H. (2020). Timely mental health care for the 2019 novel coronavirus outbreak is urgently needed. Lancet Psychiatry.

[5] Shanafelt T., Ripp J., Trockel M. (2020). Understanding and Addressing Sources of Anxiety among Health Care Professionals during the COVID-19 Pandemic. JAMA.

[6] Rivas-García F. (2020). Bioética y profesionales sanitarios en el abordaje de la pandemia provocada por COVID-19 en España. Rev. Iberoam. Bioét..

[7] O’Neill D.J. (2020). Covid-19: Clinicians need continuing professional development in ethics. BMJ.

[8] Alharbi J., Jackson D., Usher K. (2020). The potential for COVID-19 to contribute to compassion fatigue in critical care nurses. J. Clin. Nurs..

[9] Montemurro N. (2020). The emotional impact of COVID-19: From medical staff to common people. Brain Behav. Immun..

[10] Roney L.N., Acri M.C. (2018). The Cost of Caring: An Exploration of Compassion Fatigue, Compassion Satisfaction, and Job Satisfaction in Pediatric Nurses. J. Pediatr. Nurs..

[11] Stamm B. (2005). The Professional Quality of Life Scale: Compassion Satisfaction, Burnout & Compassion Fatigue/Secondary Trauma Scales.

[12] Ruiz-Fernández M.D., Pérez-García E., Ortega-Galán Á.M. (2020). Quality of life in nursing professionals: Burnout, fatigue, and compassion satisfaction. Int. J. Environ. Res. Public Health.

[13] Rodrigues H., Cobucci R., Oliveira A., Cabral J.V., Medeiros L., Gurgel K., Souza T., Gonçalves A.K. (2018). Burnout syndrome among medical residents: A systematic review and meta-analysis. PLoS ONE.

[14] Alharbi J., Jackson D., Usher K. (2020). Personal characteristics, coping strategies, and resilience impact on compassion fatigue in critical care nurses: A cross-sectional study. Nurs. Health Sci..

[15] Kim S., Kweon Y. (2020). Psychological Capital Mediates the Association between Job Stress and Burnout of among Korean Psychiatric Nurses. Healthcare.

[16] Ruiz-Fernández M.D., Ramos-Pichardo J.D., Ibáñez-Masero O., Cabrera-Troya J., Carmona-Rega M.I., Ortega-Galán Á.M. (2020). Compassion fatigue, burnout, compassion satisfaction and perceived stress in healthcare professionals during the COVID-19 health crisis in Spain. J. Clin. Nurs..

[17] Lai J., Ma S., Wang Y., Cai Z., Hu J., Wei N., Wu J., Du H., Chen T., Li R. (2020). Factors Associated With Mental Health Outcomes Among Health Care Workers Exposed to Coronavirus Disease 2019. JAMA Netw. Open..

[18] Berlinger N., Wynia M., Powell T., Micah Hester D., Milliken A., Fabi R., Jenks N.P. (2020). Source Ethical Framework for Health Care Institutions Responding to Novel Coronavirus SARS-CoV-2 (COVID-19). Guidelines for Institutional Ethics Services Responding to COVID-19 Managing Uncertainty, Safeguarding Communities, Guiding Practice.

[19] Galiana L., Arena F., Oliver A., Sansó N., Benito E. (2017). Compassion Satisfaction, Compassion Fatigue, and Burnout in Spain and Brazil: ProQoL Validation and Cross-cultural Diagnosis. J. Pain Symptom. Manag..

[20] Cohen S., Kamarck T., Mermelstein R. (1983). A global measure of perceived stress. J. Health Soc. Behav..

[21] Remor E. (2006). Psychometric properties of a European Spanish version of the Perceived Stress Scale (PSS). Span J. Psychol..

[22] Andalusian Health Service Andalusian Health Service Staff. https://www.sspa.juntadeandalucia.es/servicioandaluzdesalud/archivo-estadisticas/plantilla-del-servicio-andaluz-de-salud-3.

[23] Zhang Y.Y., Han W.L., Qin W., Yin H.X., Zhang C.F., Kong C., Wang Y.L. (2018). Extent of compassion satisfaction, compassion fatigue and burnout in nursing: A meta-analysis. J. Nurs. Manag..

[24] Powell S.K. (2020). Compassion Fatigue. Prof. Case Manag..

[25] Hernández-Padilla J.M., Ruiz-Fernández M.D., Granero-Molina J., Ortíz-Amo R., López Rodríguez M.M., Fernández-Sola C. (2020). Perceived health, caregiver overload and perceived social support in family caregivers of patients with Alzheimer’s: Gender differences. Health Soc. Care Community.

[26] Portero de la Cruz S., Cebrino J., Herruzo J., Vaquero-Abellán M. (2020). A Multicenter Study into Burnout, Perceived Stress, Job Satisfaction, Coping Strategies, and General Health among Emergency Department Nursing Staff. J. Clin. Med..

[27] Coronado-Vázquez V., Gómez-Salgado J. (2020). The error of not planning public health emergencies. Gac. Sanit..

[28] Cheng J.O.S., Ping L., Wah-Pun Sin E. (2020). The effects of nonconventional palliative and end-of-life care during COVID-19 pandemic on mental health-Junior doctors’ perspective. Psychol. Trauma.

[29] Sacco T.L., Copel L.C. (2018). Compassion satisfaction: A concept analysis in nursing. Nurs. Forum..

[30] Brito-Pons G., Librada-Flores S. (2018). Compassion in palliative care: A review. Curr. Opin. Support Palliat. Care.

[31] Li Z., Ge J., Yang M., Feng J., Liu C., Yang C. (2020). Vicarious traumatization: A psychological problem that cannot be ignored during the COVID-19 pandemic. Brain Behav. Immun..

[32] Liang Y., Chen M., Zheng X., Liu J. (2020). Screening for Chinese medical staff mental health by SDS and SAS during the outbreak of COVID-19. J. Psychosom. Res..

[33] Joob B., Wiwanitkit V. (2020). Traumatization in medical staff helping with COVID-19 control. Brain Behav. Immun..

[34] Carbone S.R. (2020). Flattening the curve of mental ill-health: The importance of primary prevention in managing the mental health impacts of COVID-19. Ment. Health Prev..

[35] Ruiz-Fernández M.D., Ortíz-Amo R., Ortega-Galán Á.M., Ibáñez-Masero O., Rodríguez-Salvador M.d.M., Ramos-Pichardo J.D. (2020). Mindfulness therapies on health professionals. Int. J. Ment. Health Nurs..

[36] Auserón G.A., Viscarret M.R.E., Goñi C.F., Rubio V.G., Pascual P.P., Galdeano E.S.D.M.G.D. (2018). Evaluation of the effectiveness of a Mindfulness and Self-Compassion program to reduce stress and prevent burnout in Primary Care health professionals. Aten. Primaria.

[37] Hevezi J.A. (2016). Evaluation of a Meditation Intervention to Reduce the Effects of Stressors Associated With Compassion Fatigue Among Nurses. J. Holist. Nurs..

